# Nutritional Status and Health Challenges Among Schoolchildren in Nepal’s Solukhumbu Valley

**DOI:** 10.3390/children12060738

**Published:** 2025-06-06

**Authors:** María Teresa Murillo-Llorente, Noemí Gil-Cuñat, Sara Moltó-Dominguez, Javier Pérez-Murillo, Manuel Tejeda-Adell, Miriam Martínez-Peris, Francisco Tomás-Aguirre, María Ester Legidos-García, Marcelino Pérez-Bermejo

**Affiliations:** 1 SONEV Research Group, Faculty of Medicine and Health Sciences, Catholic University of Valencia San Vicente Mártir, C/Quevedo No. 2, 46001 Valencia, Spain; mt.murillo@ucv.es (M.T.M.-L.); javier.perezmu@ucv.es (J.P.-M.); manuel.tejeda@ucv.es (M.T.-A.); miriam.martinez@ucv.es (M.M.-P.); paco.tomas@ucv.es (F.T.-A.); ester.legidos@ucv.es (M.E.L.-G.); 2 Department of Nursing, Faculty of Medicine and Health Sciences, Catholic University of Valencia San Vicente Mártir, C/Quevedo No. 2, 46001 Valencia, Spain; noemi.gil1@mail.ucv.es; 3 Centro de Salud de L’Eliana, Departamento Arnau de Vilanova-Lliria, 46183 Valencia, Spain; sara.molto@mail.ucv.es

**Keywords:** child undernutrition, anemia, Nepal, dietary diversity, anthropometry, blood pressure, rural health, nutritional assessment

## Abstract

Background/Objectives: Child undernutrition remains a critical public health issue in Nepal, especially in the rural district of Solukhumbu. This study aimed to assess the prevalence and clinical characteristics of undernutrition, dietary patterns, and related health indicators in school-aged children from the Shree Saraswoti Basic School in Phuleli. Methods: A descriptive, cross-sectional study was conducted between July and August 2022. Fifty-four children (51.8% boys; mean age 9.4 ± 2.1 years) were evaluated using anthropometry, clinical examination, hemoglobin measurement, and three-day 24 h dietary recall. Data were analyzed using descriptive and inferential statistics. Results: BMI z-scores indicated that 39% of children were at risk of acute undernutrition, and 2% were at risk of moderate acute undernutrition. After adjusting for altitude, 87% were classified as anemic. Diets were dominated by cereals and vegetables, with a very low intake of fruits and proteins and no dairy consumption. Dental caries affected 59% of participants. Girls presented slightly higher subcutaneous fat percentages; however, 14.8% of the children exceeded the recommended thresholds. A dietary assessment revealed poor eating habits, including excessive intake of simple carbohydrates and insufficient nutrient diversity. Although socioeconomic data were not directly collected, the findings reflect the typical context of the vulnerability of isolated mountain communities. Undernutrition indicators (BMI, clinical signs, anemia) were associated with poorer health outcomes. An unexpected moderate inverse correlation was found between BMI and both systolic (r = −0.601) and diastolic (r = −0.550) blood pressure. Conclusions: The findings reveal a high burden of undernutrition and anemia among children in Solukhumbu, linked to poor diet and structural vulnerability. Urgent, community-based interventions—including nutrition education, agricultural diversification, and improved healthcare access—are needed. Longitudinal monitoring is essential to track progress and design sustainable, multisectoral solutions.

## 1. Introduction

Child undernutrition remains a pressing public health concern in Nepal despite the country’s constitutional commitment to ensuring children’s rights to adequate food, health, and nutrition, as enshrined in the 2015 Constitution [1]. Nepal continues to record one of the highest child mortality rates globally, with an estimated 75,000 deaths annually among children—many attributed to preventable causes such as undernutrition [2]. National data from recent years reveal that 21% of children under five are underweight, and 24.4% of preschool-aged children were undernourished as of 2019 [3,4].

In the Solukhumbu Valley, particularly in the village of Phuleli, undernutrition and anemia are especially prevalent. These conditions include macronutrient deficiencies—stunting, wasting, and underweight—as well as micronutrient deficiencies, such as iron-deficiency anemia. More than one-third of children under five across Nepal are stunted, and similarly high rates of underweight and wasting persist in rural regions such as Solukhumbu [5,6]. Contributing factors include pervasive poverty, poor dietary diversity, limited healthcare access, and low agricultural productivity.

Socioeconomic status is a critical determinant of nutritional health. Families with limited financial resources are more likely to experience food insecurity, which significantly correlates with child undernutrition [7,8,9]. In Solukhumbu, where diets are heavily reliant on staple grains and market access is restricted, these vulnerabilities are heightened [8,10]. Multiple studies have emphasized the increased risk of undernutrition among children in food-insecure households, underscoring the importance of locally tailored nutrition interventions [11,12].

The Government of Nepal, through its Ministry of Health and Population, has acknowledged the severity of the undernutrition crisis. In collaboration with international agencies, it has implemented maternal education programs, food fortification initiatives, and targeted supplementation strategies [2,13]. However, systemic barriers persist, and further effort is needed to bridge gaps in service delivery and nutrition literacy.

Although Nepal lacks a formal national food guide, it employs the Food-Based Dietary Guidelines (FBDGs) developed by the Food and Agriculture Organization (FAO) and adapted to local contexts [14]. These guidelines promote dietary diversity and increased consumption of whole grains, vegetables—particularly leafy greens—legumes, lean meats, and dairy products. They also advocate limiting fat and sugar intake, using iodized salt in moderation, and engaging in physical activity. However, alignment with these recommendations remains limited, particularly in rural and mountainous areas.

According to the 2022 Nepal Demographic and Health Survey, breastfeeding practices are generally strong. Early initiation of breastfeeding occurred in 55% of children under 23 months, with 56% being exclusively breastfed up to six months [15]. Nonetheless, anemia remains prevalent among children aged 6 months to 5 years, affecting 43% of this demographic.

Iron-deficiency anemia is particularly concerning when combined with undernutrition. Although the use of iodized salt is mandatory in Nepal and has helped reduce certain cognitive and developmental issues [16], micronutrient deficiencies, especially iron deficiency, are still widespread [17]. Infections and an inadequate diet further exacerbate the situation. The socioeconomic underpinnings of anemia mirror those of undernutrition, including lack of dietary diversity, limited access to healthcare, and poor nutrition awareness [6,18]. Integrated strategies that combine nutrition education, agricultural diversification, and improved health services are essential [19,20].

Another rising concern is childhood hypertension. The Nepal Burden of Disease Report (2017) identified hypertension as the cause of nearly 14% of all deaths in the country [21]. Studies have shown that high blood pressure in children is a strong predictor of adult hypertension [22]. For this reason, the American Academy of Pediatrics recommends regular blood pressure screening starting at age three, or earlier for children at risk [23]. The interpretation of pediatric blood pressure requires the consideration of sex, age, and height percentiles [24,25].

Anthropometric indicators—such as body mass index (BMI), waist circumference, and skinfold thickness—are positively associated with blood pressure [26,27]. Nutritional status plays a vital role in maintaining healthy blood pressure levels during childhood and adolescence [28].

Despite national progress in reducing stunting (from 57% in 1996 to 25% in 2022) [15], rural disparities persist. Approximately 40% of rural Nepali children under five still fall below standard height and weight percentiles. In response, the government launched Phase II of the Multi-Sector Nutrition Program (MSNP), aligned with the Sustainable Development Goals (SDGs) [29,30]. This initiative focuses on expanding nutrition services, especially for women and children, while strengthening multisectoral coordination.

Since 2022, the Catholic University of Valencia has partnered with local institutions in Solukhumbu through development cooperation projects aimed at improving living conditions [31]. These projects prioritize child health as a vulnerable population segment. Therefore, this study was conducted at Shree Saraswoti Basic School to comprehensively assess the nutritional and health status of school-aged children in the village of Phuleli. This study adds to the existing literature by focusing on a remote, high-altitude community in Nepal (Phuleli, Solukhumbu), a setting rarely represented in national health data. The integrated field-based approach—combining anthropometry, anemia screening, blood pressure measurement, clinical signs, and dietary recall—offers a comprehensive assessment of child health in a uniquely underserved population. Specifically, we aimed to evaluate the prevalence and clinical characteristics of undernutrition, dietary patterns, and related health indicators in this vulnerable group, with the goal of generating evidence to inform future interventions and public health priorities in similarly isolated settings.

## 2. Materials and Methods

### 2.1. Study Design

This study employed a descriptive, cross-sectional, and analytical observational design. It was conducted within the framework of a development cooperation project titled “Education and Health for the Children of the Sherpa School”, aligned with the United Nations Sustainable Development Goals (SDGs) 2, 3, 4, and 5. The health assessment team included a family and community nursing specialist and a doctor in Comprehensive Nursing from the Catholic University of Valencia San Vicente Mártir (UCV), Spain.

### 2.2. Study Setting and Population

The research was conducted from 17 July to 7 August 2022, during regular school hours. The study population included all students enrolled in grades one through five at Shree Saraswoti Basic School in Phuleli village, which is located in the Solukhumbu Valley. A total population approach was used since all enrolled students were available for inclusion, eliminating the need for sampling. A total of fifty-four children participated: fourteen from grade 1 (25.9%), ten from grade 2 (18.5%), ten from grade 3 (18.5%), nine from grade 4 (16.7%), and eleven from grade 5 (20.4%). Using a full sample from a single school reflects the structure of our cooperative project and ensures full coverage of a well-defined population. However, this approach limits the generalizability of the results to other populations.

### 2.3. Ethical Considerations

The study protocol received ethical approval from the Research Ethics Committee of the Catholic University of Valencia (reference: UCV/2021-2022/189). Informed consent was obtained from the children’s parents or legal guardians. The research adhered to the ethical principles outlined in the Declaration of Helsinki.

### 2.4. Variables and Measurement Tools

The analyzed variables encompassed sociodemographic information, anthropometric measurements, clinical signs, lifestyle indicators, and dietary intake. Nutritional habits were evaluated using a three-day, 24 h dietary recall. Due to field limitations, we analyzed food group frequency rather than nutrient composition from the 24 h recall.

We conducted dietary assessments through face-to-face interviews using a multiple-pass 24 h recall method on three nonconsecutive days, including at least one weekend day, to capture variability. Each child was interviewed individually. Local interpreters and trained bilingual health staff facilitated communication and ensured cultural appropriateness. Interviewers were trained to inquire about all foods and beverages consumed at home or school, including snacks and preparation methods, to improve recall accuracy.

We compiled food consumption data from the three 24 h recalls to generate a composite dietary pattern for each participant. Instead of analyzing each day separately, we calculated the frequency of food group consumption across all three days to reflect typical intake. Based on the FAO/WHO-aligned Food-Based Dietary Guidelines (FBDG) for South Asia, foods and beverages were categorized into seven major food groups: (1) fruits, (2) vegetables, (3) grains, (4) proteins, (5) milk products, (6) miscellaneous, and (7) other. Table 1 categorizes the most frequently consumed food items by group. This grouping allowed us to assess dietary diversity and identify the prevalence of specific food groups in the children’s diets.

Anthropometric evaluations were carried out using standardized, noninvasive techniques. Height was measured using a SECA 213 portable stadiometer (measurement range: 20–205 cm; precision: 0.1 cm), with participants standing upright, barefoot, and aligned against a vertical surface. Body weight and body fat percentage were assessed using an Omron BF 508 digital scale (precision: 0.1 kg), with children dressed in light clothing. Mid–upper arm, waist, hip, and calf circumferences were measured to the nearest 0.1 cm using a SECA 201 flexible, non-elastic measuring tape. Tricep skinfold thickness was measured with a Slim Guide caliper (precision: 1 mm), following international anthropometric protocols.

Subcutaneous fat was evaluated using a Slim Guide 12-1125 skinfold caliper. Measurements included tricep, bicep, subscapular, and suprailiac skinfolds. Each site was measured three times on the non-dominant side, ensuring proper technique and reliability. The skinfold was pinched to isolate subcutaneous fat without compressing muscle tissue.

We interpreted anthropometric measurements using the WHO Child Growth Standards. Z-scores for weight-for-age, height-for-age, and BMI-for-age were calculated using the WHO AnthroPlus software. Undernutrition was defined according to the following standard thresholds: stunting (height-for-age Z-score less than or equal to –2), underweight (weight-for-age Z-score less than or equal to –2), and thinness (BMI-for-age Z-score less than or equal to –2).

Hemoglobin and hematocrit levels were determined using an EKF Hemo Control analyzer (BioBlaster S.L.), allowing for rapid field-based assessment of anemia.

### 2.5. Statistical Analysis

Descriptive and inferential statistics were used to analyze the data. Continuous variables were presented as means and standard deviations (SD), while categorical variables were reported as absolute frequencies and percentages. The Shapiro–Wilk test assessed the normality of continuous variables. Student’s t-test was applied to compare means between two groups (sex), and one-way analysis of variance (ANOVA) was used for comparisons across more than two groups (age groups). Pearson correlation coefficients were calculated to explore relationships between continuous variables. Associations between categorical variables, such as anemia status and BMI-based nutritional classification, were analyzed using the chi-square test of independence. When applicable, Fisher’s exact test was used in cases where expected cell counts were below 5.

All statistical analyses were performed using SPSS software version 23.0 (IBM Corp., Armonk, NY, USA). A *p*-value of less than 0.05 was considered statistically significant.

## 3. Results

A total of 54 students participated in the study, comprising 28 boys (51.8%) and 26 girls (48.2%). The mean age was 9.37 years (SD = 2.1), ranging from 6 to 13 years. There was no statistically significant difference in age between the sexes (*p* = 0.125). Table 2 presents the anthropometric characteristics stratified by sex.

Boys showed slightly higher values for weight, height, BMI, and circumferences, although the only statistically significant difference was found in the tricep skinfold measurement (*p* = 0.026), where girls demonstrated greater subcutaneous fat thickness.

To improve the understanding of age-related anthropometric differences, the data were analyzed by age group (6–8, 9–11, and 12–13 years) to identify developmental trends more clearly. Table 3 presents weight, height, and BMI data, which are stratified by sex and age group, to illustrate these trends more clearly.

Anthropometric data, when stratified by age group and gender, revealed consistent growth patterns across the pediatric population. As expected, weight and height increased progressively with age in both sexes. Among males, mean weight increased from 20.95 kg (SD 2.41) in the 6–8 age group to 32.39 kg (SD 5.98) in the 12–13 age group, and mean height increased from 1.16 m (SD 0.048) to 1.39 m (SD 0.082). Similar trends were observed in females, whose mean weight increased from 20.41 kg (SD 3.02) to 33.08 kg (SD 2.98) and whose height increased from 1.17 m (SD 0.066) to 1.43 m (SD 0.058) within the same age intervals. BMI values increased with age in both sexes: from 15.59 to 16.49 in males and from 14.83 to 16.28 in females. This reflects typical somatic development. While the progression was similar for both sexes, females aged 12–13 showed slightly higher mean body mass (33.08 kg vs. 32.39 kg) and taller stature (1.43 m vs. 1.39 m) than males, which may indicate early pubertal growth acceleration. Statistical analysis (ANOVA) confirmed that the differences in weight, height, and BMI among the age groups were significant for both sexes (*p* < 0.001), which justifies age stratification in the anthropometric assessments of nutritional status.

**Figure 1:**
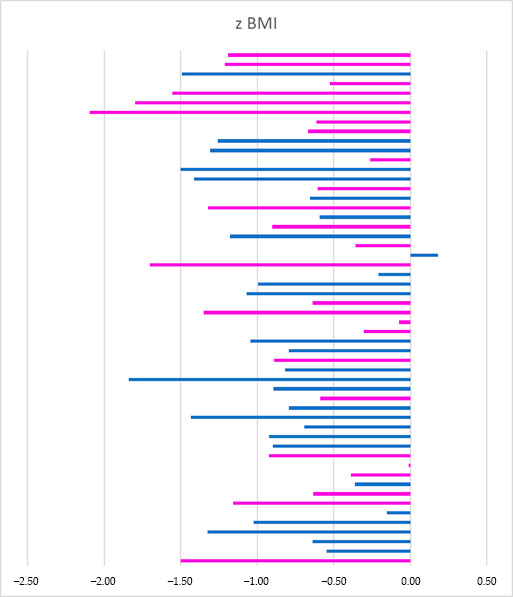
BMI z-scores of the children in the sample (*n* = 54). Blue color: boy; pink color: girl; no significant difference in the z-scores of both sexes (*p* = 0.687).

BMI z-scores revealed that 39% of the children were at risk of acute undernutrition, and 2% met the criteria for moderate acute undernutrition. Figure 1 illustrates the distribution of BMI z-scores, highlighting a clustering of values below the mean.

### 3.1. Clinical Nutritional Aspects

Table 4 shows the clinical nutritional aspects observed in the population of children studied:

Clinical evaluations revealed signs of nutritional deficiencies, including dry skin, which may be related to a vitamin A deficiency; soft nails, which may be related to an iron or protein deficiency; and thinning hair, which may be related to a zinc or biotin deficiency. These symptoms align with the typical manifestations of micronutrient deficiencies, including dry skin (14.82%), sparse hair (1.85%), soft nails (3.7%), and dry mucous membranes (3.7%). Dental caries were present in 59.26% of the children, 42.59% of whom had severe caries.

Blood pressure data by age group are presented in Table 5. Children aged 9 to 11 years had the lowest mean systolic and diastolic pressures. The unexpected finding is the elevation of DBP in the 6–8 age group.

An inverse correlation was identified between BMI and both systolic (r = −0.601, *p* = 0.017) and diastolic (r = −0.550, *p* = 0.028) blood pressure. This notable finding in malnourished populations has been reported in other low-resource contexts. In such settings, low lean body mass, reduced sympathetic activity, and chronic energy deficiency can result in paradoxical increases in blood pressure despite low BMI values. These physiological adaptations have been observed in undernourished children in Asian and African contexts, where chronic undernutrition, reduced lean mass, high carbohydrate intake, and stress may contribute to low BMI and elevated blood pressure. This is contrary to trends typically observed in pediatric populations.

Hematological assessments revealed anemia in 11.1% of children based on standard sea-level hemoglobin thresholds. However, after adjusting for the high-altitude conditions in Solukhumbu (3400 m above sea level), the prevalence of anemia rose sharply to 87%. This result underscores the importance of applying altitude-specific cutoff values. Of the 54 children evaluated, 32 (59.3%) had BMIs within the normal range, despite the high prevalence of anemia. Only one child with anemia met the criteria for moderate undernutrition, while 19 children (35.2%) were at risk of undernutrition based on BMI classification. Table 6 shows that there was no statistically significant association between anemia status and nutritional category (*p* = 0.752). This suggests that anemia in this population may not be strongly reflected in anthropometric indicators alone.

### 3.2. Dietary Patterns

Children reported consuming four daily meals: breakfast (6:30 a.m.), lunch (9:00 a.m.), an afternoon snack (1:00 p.m.), and dinner (7:00 p.m.). Breakfast typically included sweetened tea with biscuits, while lunch and dinner consisted mainly of *dal bhat*—a staple dish of rice, lentils, potatoes, and boiled vegetables. Other common items included porridge, noodles, roasted corn, and fried rice. The intake of meat, fish, and eggs was sporadic, and dairy products were entirely absent from the diet.

Dietary pattern analyses revealed that grains and vegetables were the dominant components of all meals. Although the data were collected using a three-day, 24 h dietary recall, the analysis was based on food groups rather than nutrient content due to the descriptive nature of the study and limited laboratory capacity. Fruit, protein, and especially dairy were underrepresented. For example, lunch consisted of 40% grains, 36% vegetables, 13% protein, and just 4% fruit.

**Figure 2:**
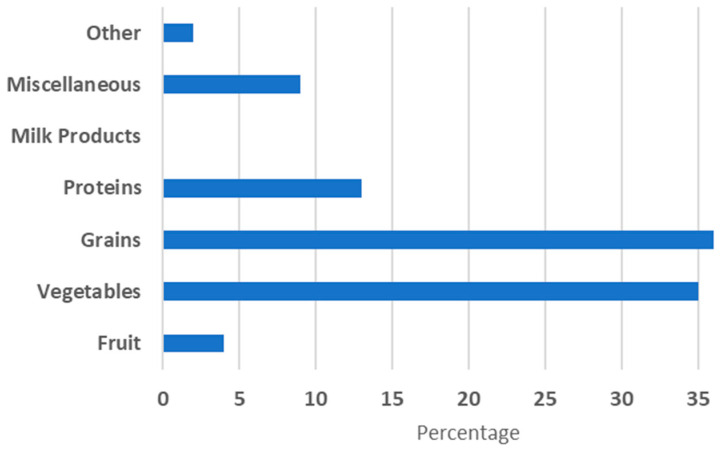
Average consumption of each food group.

Figure 2 summarizes the mean distribution of food groups across meals.

## 4. Discussion

The findings of this study underscore the multifactorial nature of child undernutrition in Nepal’s Solukhumbu Valley. The combination of limited dietary diversity, poor access to healthcare, and socioeconomic hardship contributes to suboptimal growth and development outcomes among school-aged children.

Education in the Solukhumbu Valley reflects a complex sociodemographic reality shaped by economic hardship, geographical isolation, and persistent gender disparities [31,32]. While primary education is generally accessible, secondary school attendance—especially among girls—is hindered by factors such as early marriage, menstrual stigma, and economic constraints [33,34,35]. Although these factors affect long-term health and development, they fall outside the direct scope of this study. Our findings are focused on nutritional status and health indicators observable in early childhood.

Dietary patterns in the region show significant differences from the recommendations of the Food-Based Dietary Guidelines for South Asia [14]. A recent study [36] found that minimum dietary diversity was significantly lower in rural areas than in urban areas, which is consistent with our findings of a repetitive dietary pattern. Although vegetable and cereal consumption is within the recommended ranges, indicating adequate intake of fiber, vitamins, and minerals, as well as fruit consumption, is notably low, which may have long-term health implications [37]. We highlight the high intake of lentils in the Solukhumbu diet. Previous studies have suggested that this high intake is positively associated with adequate growth, emphasizing its beneficial role in children’s diets [38,39]. Adequate protein intake has been shown to be critical in infancy, where an intake of 1.2–1.5 g/kg/day is recommended to support optimal growth and development [40]. The absence of dairy products in the diet is alarming, as they are a major source of calcium and vitamin D, which is essential for bone mineralization, especially during the rapid growth periods of childhood and adolescence [37,41]; it should be noted that rural Nepal has significant vitamin D deficiencies [42].

In addition, tea consumption, which is mostly part of what we have termed “miscellaneous”, has antioxidant benefits due to its high polyphenol content [43]. However, there are concerns about its effects on childhood iron absorption, a mineral critical for growth and development [44]. Children also tend to consume large amounts of added sugar from tea, which may contribute to health problems such as dental caries [45]. The American Academy of Psychiatry advises against caffeine consumption before the age of 12 due to a lack of evidence regarding its safety [46], and tea contains caffeine.

To detect signs of undernutrition, a thorough physical examination, including inspection of factors such as hair, mucous membranes, nails, teeth, and skin, is essential [47]. In our study, abnormalities were found, particularly in mucous membranes and dental health. The relationship between oral hygiene habits and diet is crucial, as undernutrition can aggravate the occurrence of dental caries in children [48]. In addition, the excessive consumption of fermentable carbohydrates in children’s diets contributes to this problem, and the lack of dairy products in their diets aggravates the situation, as these have a protective effect against caries [49].

Sex-specific anthropometric findings were consistent with the expected developmental patterns. Girls had higher subcutaneous fat in the triceps region, while boys were taller and heavier overall. These differences reflect known sex-based growth trajectories during childhood [50]. This is crucial in interpreting the increasing incidence of undernutrition and obesity in this region. It is noteworthy that the relationship between BMI and age is similar to that found in rural Tanzania, where a higher age index corresponds to a higher BMI [51]. However, in this study, we found that boys had a higher BMI than girls, which contradicts previous observations. Overall, our analysis shows that all children had a below-average BMI with a high number of cases of undernutrition, which is consistent with other work [52].

The relationship between BMI and BP among children in Nepal appears to be more complex than a simple direct correlation, as we found a negative relationship between BMI and BP, contrary to expectations, suggesting the need to investigate not only the effect of obesity on BMI [53] but also the effect of undernutrition on BMI. Several physiological, environmental, and genetic factors may contribute to this complex association, including dietary habits, physical activity levels, and the culture of Nepalese society. An important aspect influencing the negative association between BMI and blood pressure in the children studied may be the mix of dietary and socioeconomic factors with lifestyle. Many children, especially those in disadvantaged rural areas, often lack access to a nutritious diet and may rely heavily on high-carbohydrate, low-fat foods, which may lead to lower BMIs with variable blood pressure responses [54,55].

Socioeconomic status may have an even greater impact on blood pressure than BMI alone [56,57]. Additionally, elevated dietary sodium intake, often due to the consumption of packaged soups or noodles, and psychosocial stress in low-income children may contribute to abnormal blood pressure, even in underweight individuals [58,59]. Shakya and Bajracharya suggested that children classified as normal weight or underweight, as nearly all of the children in this study were, may suffer from hypertension due to factors such as high dietary sodium intake or other stressors [60,61,62]. These findings suggest possible disparities in access to healthcare and nutrition education in Nepal, where children from lower-income households may be at greater risk for health problems despite having lower BMIs.

The prevalence of anemia in our population is significantly lower than the national average. Similar results were found in a Nepalese study, in which anemia was more associated with altitude and corresponding hematologic adaptations [63]. These and other studies suggest that while adjusting for altitude is important, looking for other markers, such as ferritin and hepcidin, may provide more information for diagnosing iron deficiency anemia. Although they were not included in our current analysis, we propose including them in future research projects conducted in similar settings. Similarly, future studies using regression models are needed to explore the statistical relationships between food group intake and nutritional outcomes.

### Limitations

This study has several limitations. First, language barriers may have influenced the accuracy of self-reported dietary intake. To minimize this, trained local interpreters assisted with data collection, and the materials were reviewed by bilingual health professionals. Second, this study included a small, non-randomized, and geographically localized sample from a single school, which may introduce selection bias and limit the generalizability of the findings to other rural or urban populations. Third, anemia diagnosis was based on a single-point hemoglobin measurement, without complementary biomarkers such as ferritin or hepcidin, which restricts interpretation and prevents accurate differentiation between anemia types. Additionally, the lack of a control group or comparative site limits the contextualization of the results. Despite these limitations, the study’s strengths include complete coverage of the accessible population, standardized anthropometric and clinical procedures, and multi-day direct dietary observation, offering a comprehensive view of child health in a high-altitude, underserved setting.

## 5. Conclusions

Despite the majority presenting with BMI values within normal ranges, this study identified a high prevalence of anemia and clinical signs of micronutrient deficiencies in school-aged children from rural Solukhumbu, Nepal. These results highlight the limitations of using anthropometric measures alone to evaluate the nutritional status of vulnerable populations. Poor dietary diversity and inadequate intake of protein- and vitamin-rich foods, as well as signs such as extensive dental caries and skin alterations, reflect underlying nutritional deficiencies that BMI alone does not capture.

In the context of a high altitude and limited access to healthcare and diverse foods, future research should incorporate biochemical assessments and explore the role of socioeconomic and environmental factors in shaping children’s nutritional health. Public health interventions should focus on improving nutrition education, promoting access to iron- and vitamin-rich foods, and implementing routine clinical screenings that go beyond standard growth indicators.

International collaboration through development projects, such as those led by the Catholic University of Valencia, provides an effective model for advancing health equity. These initiatives should be expanded and sustained to support long-term improvements in nutrition and overall child well-being in underserved regions like Solukhumbu. By addressing these challenges comprehensively, it is possible to promote healthier growth trajectories and improve the quality of life for vulnerable children in rural Nepal.

These findings call for actionable, community-based strategies, including nutritional education tailored to local realities, improved access to micronutrient-rich foods, and regular clinical screening in school settings. Future studies should include longitudinal follow-up and consider integrated interventions involving families, schools, and local health authorities to sustainably address the multifactorial burden of undernutrition.

## Figures and Tables

**Table 1:** Food grouping used in dietary pattern analysis.

Groups	Food
Fruit	Passion Fruit Banana
Vegetables	Potato (baked/chipped) Corn (cooked/roasted) Cucumber Celery
Grains	Rice Lentils Crackers Noodles (prepared Chinese noodles)
Proteins	Meat Fish Egg
Milk Products	-
Miscellaneous	Tea and, in the minority, fats
Other	Oil: rice oil Herbs and spices: citronella Free sugars: honey

**Table 2:** Anthropometric characteristics of the study sample stratified by sex (*n* = 54).

	Sex	Mean	SD *	*p*-Value **
Weight (Kg)	Total	25.66	5.94	
	Male	26.69	6.03	0.185
	Female	24.54	5.74	
Height (m)	Total	1.27	0.111	
	Male	1.28	0.108	0.307
	Female	1.25	0.113	
BMI (Kg/m^2^)	Total	15.79	1.31	
	Male	16.06	1.24	0.118
	Female	15.50	1.35	
Waist circumference (cm)	Total	59.26	4.96	
	Male	60.46	5.15	0.066
	Female	57.98	4.49	
Hip circumference (cm)	Total	67.94	6.11	
	Male	68.27	6.01	0.687
	Female	67.59	6.32	
Arm circumference (cm)	Total	20.51	2.69	
	Male	20.44	3.40	0.853
	Female	20.58	1.68	
Calf circumference (cm)	Total	27.93	2.75	
	Male	28.40	2.98	0.200
	Female	27.43	2.43	
Subscapular skinfold (mm)	Total	7.43	2.67	
	Male	7.21	2.60	0.550
	Female	7.65	2.77	
Suprailiac skinfold (mm)	Total	6.21	2.73	
	Male	5.70	2.73	0.151
	Female	6.77	2.67	
Bicep skinfold (mm)	Total	5.81	2.26	
	Male	5.55	1.73	0.382
	Female	6.10	2.72	
Tricep skinfold (mm)	Total	9.03	2.51	
	Male	8.30	2.40	0.026
	Female	9.81	2.43	

* SD: Standard Deviation; ** Unpaired Student *t*-Test *p* < 0.05; BMI: Body Mass Index.

**Table 3:** Anthropometric characteristics of the sample stratified by sex and age group (*n* = 54).

	Sex	Age	Mean	SD *	*p*-Value **
Weight (Kg)	Male	6–8	20.95	2.41	<0.001
		9–11	25.90	4.27	
		12–13	32.39	5.98	
	Female	6–8	20.41	3.02	<0.001
		9–11	25.30	3.74	
		12–13	33.08	2.98	
Height (m)	Male	6–8	1.16	0.048	<0.001
		9–11	1.27	0.069	
		12–13	1.39	0.082	
	Female	6–8	1.17	0.066	<0.001
		9–11	1.26	0.060	
		12–13	1.43	0.058	
BMI (Kg/m^2^)	Male	6–8	15.59	0.76	0.046
		9–11	16.02	1.45	
		12–13	16.49	1.11	
	Female	6–8	14.83	1.14	<0.001
		9–11	15.97	1.29	
		12–13	16.28	1.38	

* SD: Standard Deviation; ** ANOVA Test *p* < 0.05; BMI: Body Mass Index.

**Table 4:** Clinical–nutritional aspects of the children under study (*n* = 54).

Clinical Parameter	Observed Condition	*n* (%)
Skin condition	Good	45 (83.33)
	Dry	8 (14.82)
	Dehydrated	1 (1.85)
Hair condition	Good	53 (98.15)
	Sparse	1 (1.85)
Condition of nails	Normal	52 (96.30)
	Soft	2 (3.70)
Condition of mucous membranes	Moisturized	52 (96.30)
	Bad condition	2 (3.70)
Condition of dentition	Good condition	22 (40.74)
	Light caries/stains	9 (16.67)
	Abundant caries	23 (42.59)

**Table 5:** Systolic and diastolic blood pressure values by age range.

	Age Range (Years)	*n*	Mean	SD	95% Confidence Interval	
					Lower Limit	Upper Limit
Systolic Blood Pressure (mmHg)	6–8	18	110.83	14.17	103.79	117.88
	9–11	23	107.35	12.11	102.11	112.58
	12–14	13	112.15	14.80	103.21	121.10
	Total	54	109.67	13.39	106.01	113.32
Diastolic Blood Pressure (mmHg)	6–8	18	75.06	10.46	69.85	80.26
	9–11	23	70.65	10.83	65.97	75.33
	12–14	13	72.85	10.44	66.54	79.15
	Total	54	72.65	10.59	69.76	75.54

**Table 6:** Distribution of BMI-based nutritional categories according to anemia status after altitude adjustment (*n* = 54).

Anemia	Moderate Undernutrition	At Risk of Undernutrition	Normal	*p*-Value *
No	0	2	5	0.752
Yes	1	19	27	

* Chi-Square Test *p* < 0.05.

## Data Availability

The raw data supporting the conclusions of this article will be made available by the authors on request due to legal restrictions regarding data on minors.

## Abbreviations

The following abbreviations are used in this manuscript:

FBDG	Food-Based Dietary Guidelines
FAO	Food and Agriculture Organization of the United Nations
HTN	Hypertension
BP	Blood Pressure
SBP	Systolic Blood Pressure
DBP	Diastolic Blood Pressure
WC	Waist Circumference
MSNP	Multi Sector Nutrition Program
SDG	Sustainable Development Goals
UCV	Catholic University of Valencia
